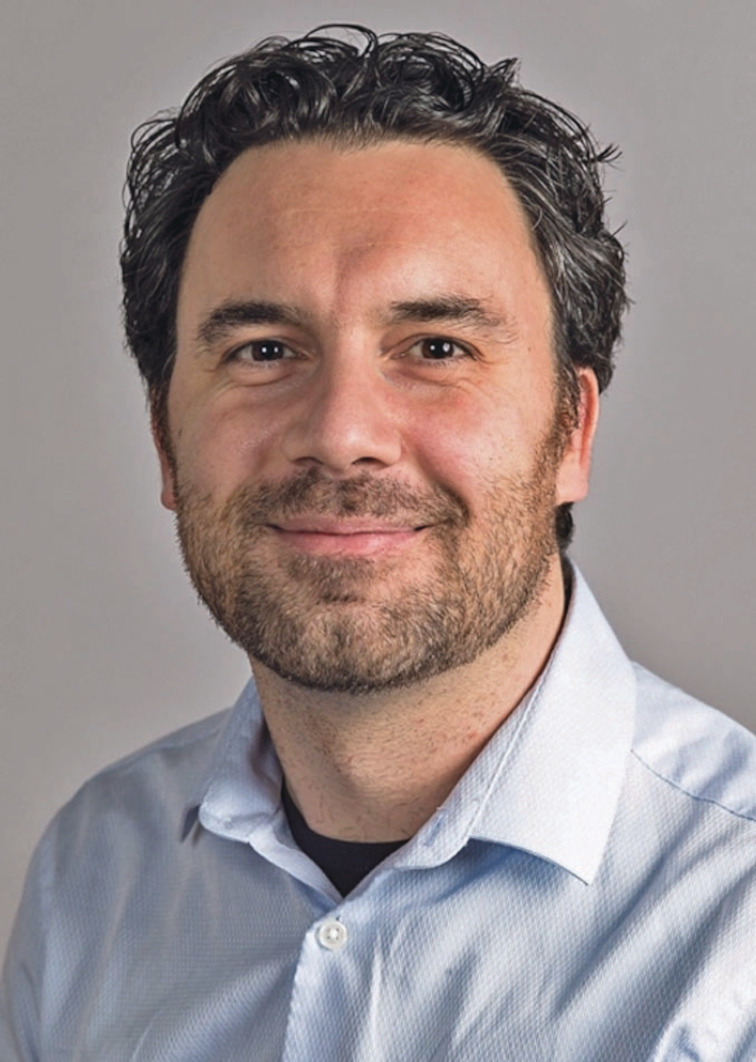# G‐Quadruplexes as Key Transcriptional Regulators in Neglected Trypanosomatid Parasites

**DOI:** 10.1002/cbic.202300265

**Published:** 2023-05-23

**Authors:** Ludovica Monti, Marco Di Antonio

**Affiliations:** ^1^ Chemistry Department, Imperial College London Molecular Sciences Research Hub 82 Wood Lane W12 0BZ London UK; ^2^ The Francis Crick Institute 1 Midland Road NW1 1AT London UK; ^3^ The Institute of Chemical Biology Molecular Sciences Research Hub 82 Wood Lane W12 0BZ London UK

**Keywords:** DNA secondary structures, G-quadruplex, kinetoplastid parasites, transcriptional regulation, Trypanosomatids

## Abstract

G‐quadruplexes (G4s) are nucleic acid secondary structures that have been linked to the functional regulation of eukaryotic organisms. G4s have been extensively characterised in humans and emerging evidence suggests that they might also be biologically relevant for human pathogens. This indicates that G4s might represent a novel class of therapeutic targets for tackling infectious diseases. Bioinformatic studies revealed a high prevalence of putative quadruplex‐forming sequences (PQSs) in the genome of protozoans, which highlights their potential roles in regulating vital processes of these parasites, including DNA transcription and replication. In this work, we focus on the neglected trypanosomatid parasites, *Trypanosoma* and *Leishmania* spp., which cause debilitating and deadly diseases across the poorest populations worldwide. We review three examples where G4‐formation might be key to modulate transcriptional activity in trypanosomatids, providing an overview of experimental approaches that can be used to exploit the regulatory roles and relevance of these structures to fight parasitic infections.

## Graphical abstract

In this concept article, we discuss the potential roles of DNA G‐quadruplexes (G4s) secondart structures, in modulating the transcriptional control of neglected trypanosomatid parasites, which cause deadly diseases in the poorest populations worldwide. We provide an overview of experimental approaches that can be used to exploit the regulatory roles and relevance of G4s to fight parasitic infections.

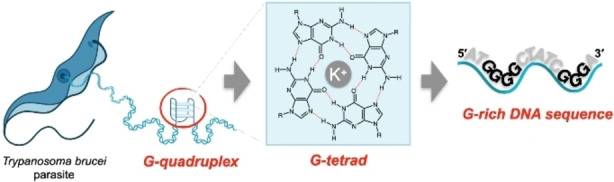

## 1 Introduction

Trypanosomatids are single‐cell protozoan parasites that cause severe human diseases. They are spread worldwide but endemic in the poorest and most vulnerable populations in Central and South America, and in various regions of Africa, where treatment and prevention are often neglected.^[1]^ These parasites are mostly transmitted by insect vectors to different hosts, including humans and both wild and domesticated animals,^[2]^ thus presenting a serious threat to health, society, and economics.^[3]^ Chagas disease (also known as American trypanosomiasis) is caused by the agent *Trypanosoma cruzi*, whilst human African trypanosomiasis (HAT; also known as African sleeping sickness) is caused by *Trypanosoma brucei gambiense* and *Trypanosoma brucei rhodesiense*, and visceral and cutaneous leishmaniasis are caused by parasites of the genus *Leishmania*.^[4]^ These diseases are debilitating and can be fatal without treatment. Despite encouraging progress in the development of effective and safer therapeutics and the few new drugs in the pipeline for clinical trials,^[5]^ the threat of resistance against the limited number of drugs currently available is a serious concern that requires immediate intervention.^[6]^ A summary of current treatments used for these kinetoplastid infections is reported in Table 1. In an effort to identify novel drug targets, G‐quadruplexes (G4s) have received considerable attention in the last decade.^[7]^

**Table 1:** Drug treatments currently used to treat kinetoplastid infections caused by *Trypanosoma cruzi*, *Trypanosoma brucei*, and *Leishmania* spp. parasites.

Disease	Drug	Agent	Efficacy	Route of administration
Chagas disease	Benznidazole	*Trypanosoma cruzi*	Acute (asymptomatic or mild) and chronic (cardiac and digestive disease) stages	Oral
	Nifurtimox			Oral
Human African trypanosomiasis	Pentamidine	*Trypanosoma brucei gambiense*	Acute (haemolymphatic stage)	Intramuscular injection
	Suramin	*T. b. rhodesiense*	Acute stage	Intravenous injection
	Nifurtimox – eflornithine (NECT)	*T. b. gambiense*	Chronic (neurological stage)	Intravenous injection
	Fexinidazole	*T. b. gambiense*	Acute and chronic stages	Oral
	Melarsoprol	*T. b. rhodesiense*	Chronic stage	Intravenous injection
Visceral (VL) and cutaneous leishmaniasis	Pentavalent antimonials	*Leishmania donovani, L. infantum, L. tropica,* *L. aethiopica, L. major, L. mexicana, L. amazonensis,* *L. braziliensis, L. guyanensis*	First‐line treatment; variable efficacy depending on countries	Intravenous or intramuscular injection
	Miltefosine		Effective for VL in India; ineffective as single dose in Asia and Africa; not registered in many endemic countries	Oral
	Amphotericin B		Effective for VL in India	Intravenous injection
VLleishmaniasis	Paromomycin	*L. donovani, L. infantum*	Effective in India and Africa	Intramuscular injection

**Figure 1:**
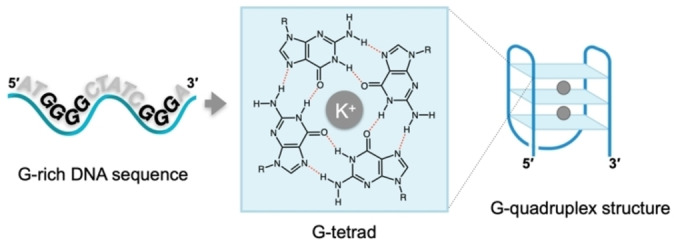
Schematic representation of a DNA G‐quadruplex structure.

G4s (Figure 1) are non‐canonical DNA or RNA structures formed under physiological conditions by the stacking of guanine tetrads (G‐tetrad). In G‐tetrads, (G)‐rich sequences are arranged through G‐G Hoogsteen base pairing and further stabilised by the presence of a central monovalent cation (increased G4‐stability: K^+^>Na^+^≫Li^+^).^[8]^ These structures are found throughout the genome of all eukaryotic species where they have been proposed to play essential roles in maintaining cellular homeostasis.^[9]^ In the human genome, G4s are enriched at gene promoters, telomeres, and transcription factor binding sites,^[10]^ highlighting their potential role in the regulation of gene‐expression and their prospective to be leveraged as therapeutics.^[11]^

**Figure 2:**
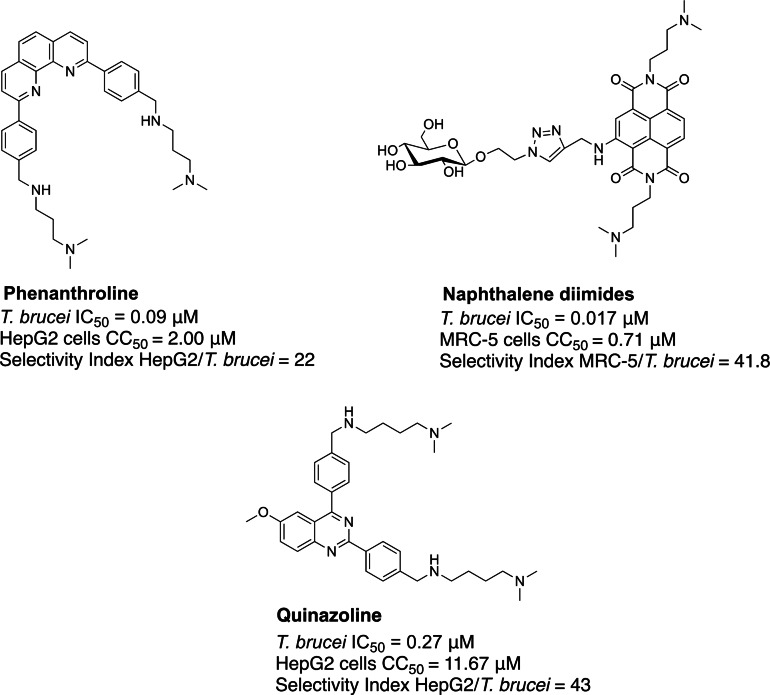
Representative examples of G4‐ligands with reported anti‐trypanosomal activity and selectivity: 2,9‐bis[(substituted‐aminomethyl)phenyl]‐1,10‐phenanthroline,^[18c]^ carbohydrate conjugated naphthalene diimide,^[16a]^ and 2,4‐bis[(substituted‐aminomethyl)phenyl]quinazoline.^[18e]^

Computational analyses and *in vitro* experiments have confirmed the presence of putative G‐quadruplex forming sequences (PQS) in many and varied infectious organisms, including bacteria,^[12]^ viruses,^[13]^ helminths,^[14]^ and parasites such as *Plasmodium falciparum* (the causative agent of malaria),^[15]^ and a number of trypanosomatid species (*e. g*., *T. brucei*, *T. cruzi*, *L. major*).^[16]^ These studies revealed a high abundance of PQS in kinetoplastid parasites compared to other parasitic agents. For example, using the G4Hunter predictive algorithm, Lombardi *et al*. identified over 100,000 and 29,000 PQS in the genomes of *L. major* and *T. brucei*, respectively.^[16c]^ This can be represented as a frequency of 3.1 and 0.81 PQS per kb of the *L. major* and *T. brucei* genomes, which is comparable to the frequency of PQS in the human genome, at 0.9 PQS/kb; and highlights that trypanosomatid parasites might also carry a significant prevalence of G‐stretches that could potentially assemble into G4s. In 2019, the Balasubramanian group was the first to experimentally demonstrate that predicted PQS could indeed form within the genome of different species, including *T. brucei* and *L. major*.^[17]^ The authors generated genomic maps of G4s through adaptation of the Illumina sequencing platform to specifically map the distribution of observed G‐quadruplexes (OQs) on a genomic scale. Briefly, Marsico *et al*., sequenced twice the genomic material changing sequencing buffers to extract structural information about DNA. Firstly, they sequenced genomic DNA under conditions that do not stabilise G4s (*i. e*., Li^+^ buffer), and successively the same material was sequenced again under conditions that stabilise G4s (*i. e*., K^+^ buffer or G4‐ligands). The difference (mismatch) in sequencing reads between the two conditions was used as an indication of G4‐formation. These studies revealed that a significant amount of the OQs found in *T. brucei* were located in 5′UTR regions (*i. e*., 44 %=1,413) or gene promoters (36 %=1,175). Conversely, in *Leishmania*, 12 % and 14 % of total OQs (*i. e*., 2327 over 16,988) were in 5′UTR and gene promoters, respectively. Interestingly, the G4 distribution pattern in *Trypanosoma* parasites is similar to that found in the genomes of higher order level and phylogenetically distant mammals, like humans and mice. These findings reinforce the hypothesis that G4s might play important regulatory roles in the biology of these protozoan pathogens, especially gene expression and transcriptional regulation. Interestingly, a number of recent papers describe the discovery and development of G4‐ligands with anti‐parasitic activity.[^16a^, ^18^] Among the different molecular scaffolds, 2,9‐bis[(substituted‐aminomethyl)phenyl]‐1,10‐phenanthroline,[^18c^, ^18g^] napthalene diimide,[^16c^, ^18d^, ^18f^, ^18h^, ^18i^] and quinazoline^[18e]^ derivatives have been reported to show promising *in vitro* anti‐trypanosomal activity and selectivity (Figure 2).

In this perspective article, we highlight three interesting biological functions of trypanosomatid parasites that have been linked to G4‐formation and that might be exploited to develop new anti‐parasitic drugs. Finally, we provide an overview of experimental approaches and future directions that could be explored to further characterise and validate the biological relevance of G4s in trypanosomatid parasites.

## 2 G4s As Modulators of Kinetoplastid DNA Replication

**Figure 3:**
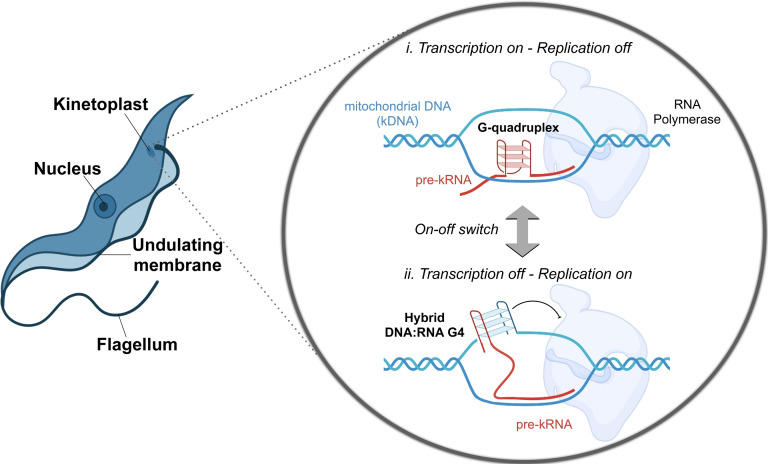
Schematic representation of the proposed mechanism of G‐quadruplexes (G4s) in the modulation of kinetoplastid DNA (kDNA) transcription and replication on‐off switch.

Trypanosomatid parasites are characterised by the presence of a single mitochondrion, which contains the kinetoplast where the mitochondrial DNA (kDNA) is stored. The kDNA is formed by two types of circular DNA molecules: the minicircles, which are a heterogenous population of thousands of guide (g)RNA‐encoding molecules of 0.5–10 kb in length; and the maxicircles, which are identical copies of DNA of approximately 20–40 Kb in length.^[19]^ Within the kinetoplast, the editosome – an ∼20S multi‐protein complex – regulates the transcription of the kDNA into mature kRNA by allowing the pre‐kRNA to bind to the template gRNA and undergo an editing process (known as pan‐editing) during which hundreds of uracil‐nucleotides are inserted and/or deleted.^[20]^ Leeder *et al*. used bioinformatic analysis and *in vitro* reverse transcriptase (RT) stop assays to characterise G4‐formation in G‐rich pre‐kRNA of *T. cruzi*, *T. brucei*, and *L. tarantolae*.^[21]^ The authors demonstrated that up to 27 G4s can be detected in the pre‐kRNA (Figure 3, *i. Transcription on – Replication off*). Half of these G4s are unwound by the editosome during the transcriptional process, thus facilitating the formation of intermediate pre‐kRNA‐gRNA. Once the intermediate is formed, it can then be converted into functional kRNA.

In addition, the authors elegantly present a theory where G4s act as key regulators of the kDNA maxicircle replication in trypanosomatids. Briefly, it is known that G4 can form hybrid DNA:RNA G4 structures between the non‐template DNA and newly‐synthesised RNA,^[22]^ therefore, the authors hypothesised that the formation of these structures would create a physical obstacle, thus leading to a stop in transcription to favour replication (Figure 3, *ii. Transcription off – Replication on*). The work from Leeder *et al*.,^[21]^ therefore provides additional evidence that G4‐structures are not random tri‐dimensional DNA structures that act as obstacles to physiological cellular functions, but they indeed mediate and finely regulate key processes involved in the parasite's life cycle.

Furthermore, the ability of G‐rich sequences to form inter‐molecular G4s represents a significant structural feature that highlights the need for additional studies to reveal the role of G4s when formed in a unimolecular or multimolecular fashion. As evidenced by Leeder *et al*., ^[21]^ the ability of G4s to form inter‐molecular structures within the transcribed RNA strand can act as a transcriptional activator and a replication repressor (Figure 3). This highlights how the dynamic formation of different G4s, such a hybrid DNA:RNA G4 or a RNA G4 formed in the transcribed RNA (Figure 3), can be leveraged by the parasite to modulate transcriptional activity. Therefore, the selective molecular targeting of hybrid DNA:RNA G4s in this context, could potentially be used to repress transcription; whilst promoting the RNA G4‐formation could instead favor transcription (Figure 3). These complex dynamics between the formation of mutually exclusive G4s emphasize how the development of chemical probes that selectively target inter‐molecular G4s^[23]^ or RNA G4s^[24]^ could be employed in the future to modulate parasite transcription in a rationally designed fashion.

Finally, it has been demonstrated that the drug diminazene aceturate (berenil), which is used to treat animal trypanosomiasis, hampers the structure and function of mitochondria in *T. cruzi*.^[25]^ Interestingly, this compound is known to bind G4 structures with high selectivity (*i. e*., *in vitro* nanomolar dissociation constant).^[26]^ However, the characterisation of the binding affinity of berenil for G4s has been validated through biophysical methods only, such as UV and NMR based approaches, and using G4‐forming oligonucleotides derived from human genomic regions (such as c‐Myc, c‐kit1).^[26]^ There is therefore a knowledge gap in understanding the mode of action of berenil *in vivo* in trypanosomatids. In the future, it will be worth exploring if berenil can bind to inter‐molecular G4s and prevent the editosome from resolving these structures, leading to transcriptional stalling. Similarly, the binding affinity of berenil for inter‐molecular G4s to inhibit kDNA replication by physically impeding RNA polymerases from processing the template strand needs to be validated.

It is becoming increasingly evident that inter‐molecular G4s can form in living organisms, linking distal DNA regions by G‐G base pairing.^[23]^ For example, the recent discovery of a human protein that selectively recognises inter‐molecular *versus* intra‐molecular G4s^[27]^ suggests that these distal G4s may form in humans. The studies reported above in *T. cruzi* also seem to suggest the relevance of inter‐molecular G4s in parasites, thus confirming the increasing evidence that supports their potential roles in DNA biology.

## 3 G4s As Transcriptional Regulators of Epigenetic Modifications

**Figure 4:**
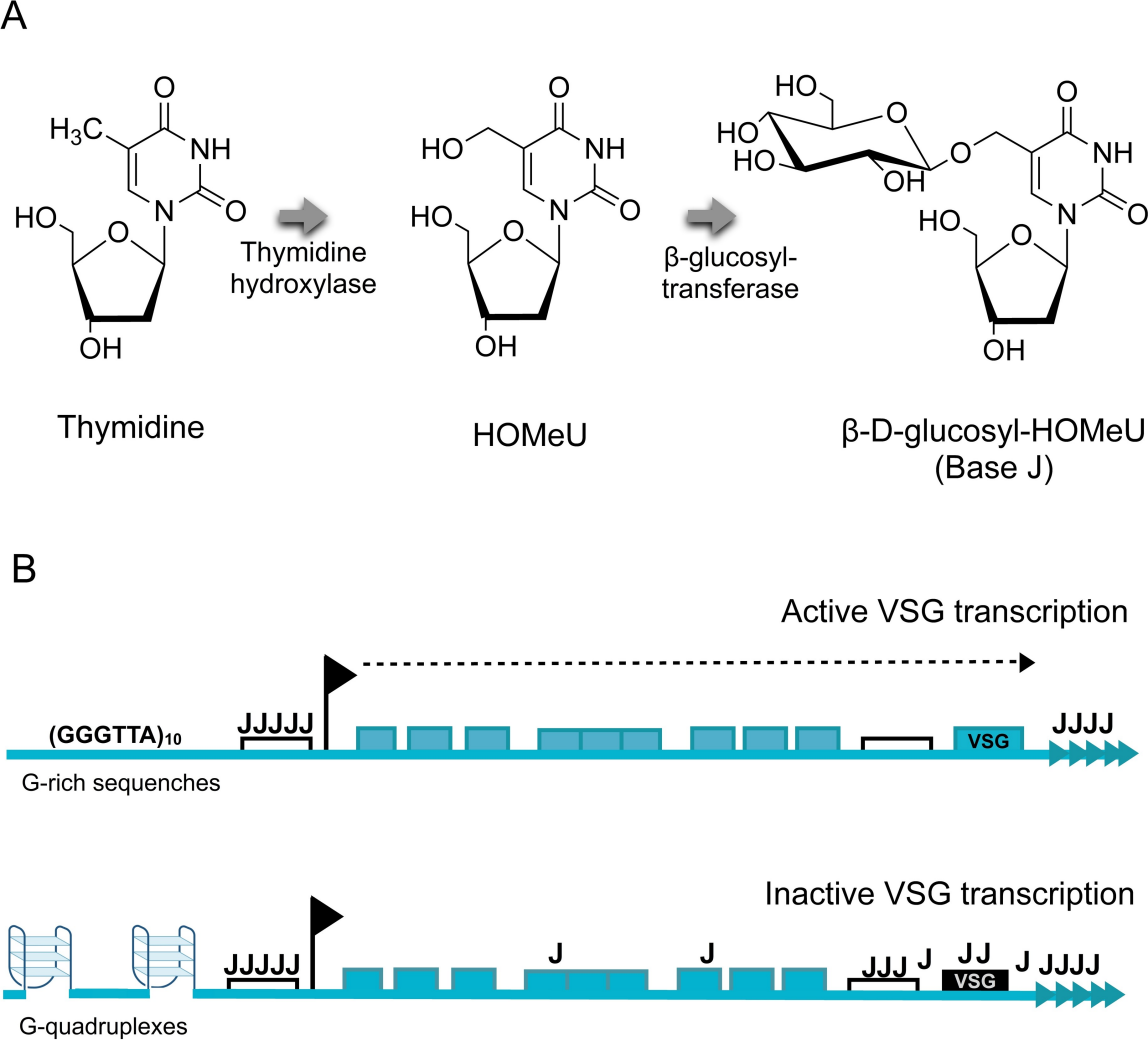
A) Biosynthesis of β‐D‐glucosyl‐hydroxymethyluracil (HOMeU) or base J. B) Schematic representation of the hypothesised mechanism of G‐quadruplexes as modulators of base J insertion. Blocks indicate expression site associated genes.

*Base J* or β‐D‐glucosyl‐hydroxymethyluracil is an epigenetic modification that occurs uniquely in kinetoplastid organisms, including parasites of the trypanosomatid clade. During this process, up to 1 % of thymidine (T) nucleotides are replaced by J bases.^[28]^ The biosynthesis of base J occurs in two steps: the first step is catalysed by two thymidine hydroxylase enzymes, JBP1 and 2, which oxidizes a specific DNA thymidine to hydroxymethyluracil (HOMeU); the second step involves a β‐glucosyl‐transferase that converts HOMeU to base J by adding glucose at the hydroxylated site (Figure 4A). The resulting base modification has different transcriptional functions, many of which remain unknown. In trypanosomes, base J is a marker of gene silencing of the Variant Surface Glycoprotein (VSG) expression sites by preventing the modified DNA from being recognised and cleaved by restriction enzymes.^[29]^ This modification is site‐specific and it is primarily found in 99 % of telomeric (GGGTTA)_n_ repeats and in regions where transcription starts and stops.^[30]^ By using Single Molecule, Real‐Time (SMRT) sequencing, Genest *et al*.^[31]^ demonstrated that the J modification occurs at specific insertion sites (identified as ‘entry’ sequences, T(N)_12_A) that are close to G‐rich sequences. These G‐rich flanking sequences near J insertion sites are characterised by 3–4 consecutive runs of Gs, suggesting a potential to form G4 structures. The authors, therefore, speculate that the presence of G4s at the entry sites might act as mediators for J site identification and/or spreading by JBP1 and 2 enzymes (Figure 4B). Although this hypothesis is yet to be validated, it would align with previous observations on the human DNA‐methyl transferase1 (DNMT1), which indicated that this epigenetic modifier enzyme can bind to G4s and prevent DNA‐methylation in nearby regions.^[32]^

To address this, there is the need to better understand G4‐distribution throughout the parasites genomes and, ideally, throughout their different life‐cycle stages. An example approach involves generating a reference G4 map using chromatin immuno‐precipitation with a G4‐specific antibody, followed by high‐throughput sequencing (G4 ChIP‐seq), as previously described in human cells.^[33]^ ChIP‐seq is performed using paraformaldehyde‐fixed cells and allows to gain a view of G4‐formation in the context of native chromatin. However, ChIP‐seq might be a challenging technique to push forward in this context, given that chromatin extraction requires a high number of cells (millions), and the sonication step to obtain chromatin fragments of appropriate size might require significant optimisation. Therefore, an alternative approach to obtain a G4 map could be Cleavage Under Targets and Tagmentation (CUT&Tag), which requires a lower number of cells (thousands) and chromatin fragmentation is achieved *in situ*.^[34]^ The ChIP‐seq CUT&Tag approaches can be coupled with data from SMRT sequencing to facilitate a detailed investigation of the presence of G4‐forming sequences at specific genomic sites. Here, an overlap of G4 *loci* and base J modifications would indicate that G4s can act as epigenetic mediators with potential impact on transcriptional regulation of key genes, such as VSG, in trypanosomatid parasites.

## 4 G4‐Mediated Antigenic Variation and Virulence Control

To survive in the host bloodstream, *Trypanosoma brucei* parasites evade immune responses using a dense glycoprotein coat, the VSG, which undergoes periodic antigenic switching to allow sustained and prolonged infections. Expression of the VSG gene is highly dependent on the transcriptional activity of RNA Pol I. Without a functional Pol I, trypanosomes are not able to survive in the mammalian host.^[35]^

**Figure 5:**
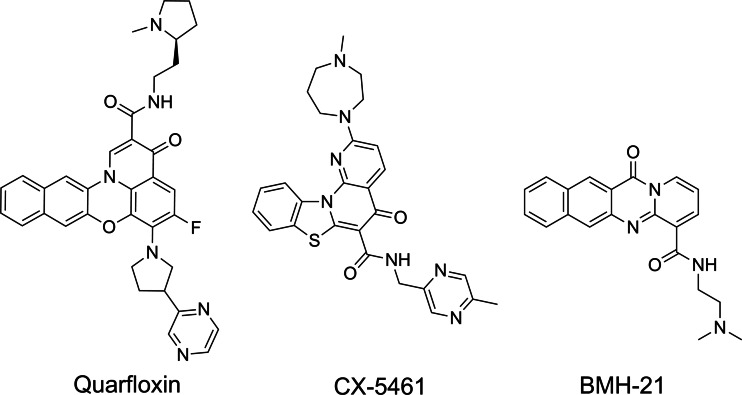
Chemical structures of quarfloxin, CX‐5461, and BMH‐21.

**Figure 6:**
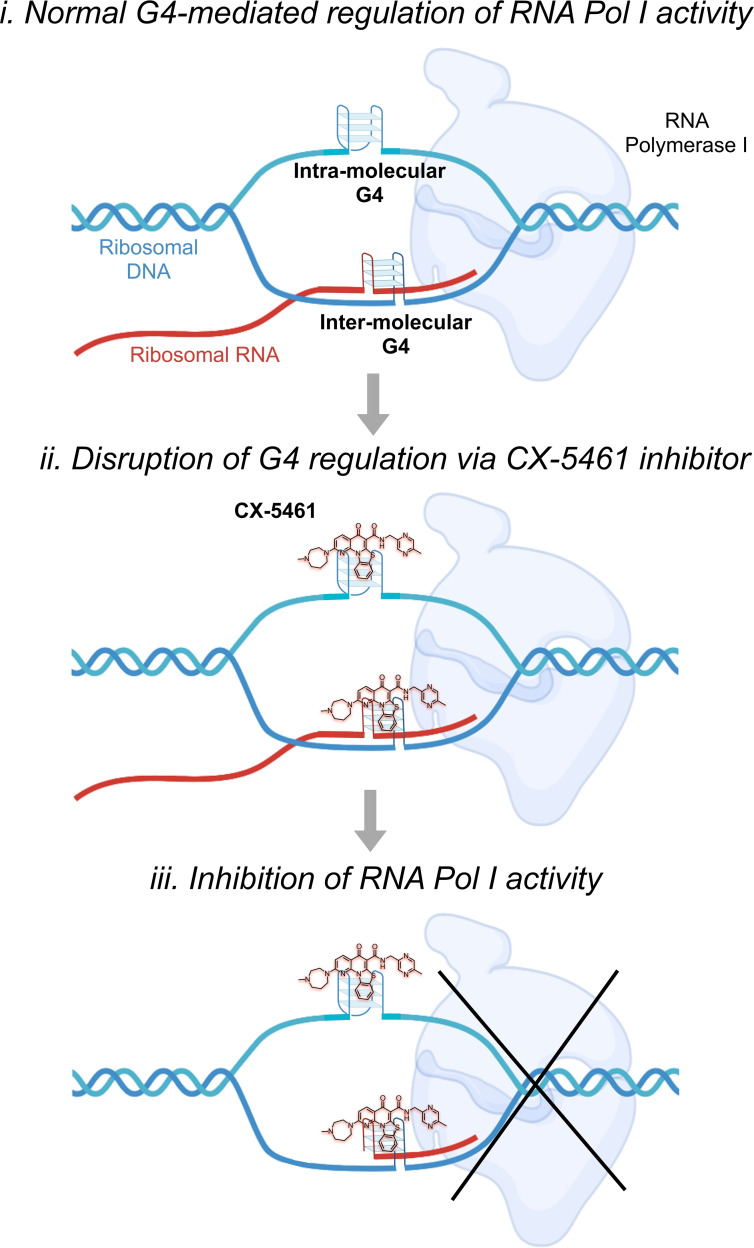
Schematic representation of potential inhibition of the transcriptional function of RNA Pol I as mediated by the G‐quadruplex ligand, CX‐5461.

The Rudenko group investigated the anti‐parasitic activity of three well‐characterised anticancer compounds that are known to inhibit the activity of Pol I – quarfloxin, CX‐5461, and BMH‐21 (Figure 5).^[36]^ These compounds exhibited potent killing activity against *T. brucei* parasites and reduced toxicity against human cells. In *T. brucei*, these compounds cause a significant reduction (>80 %) in levels of ribosomal RNA and VSG221 precursor transcripts, however, their exact mechanism of Pol I inhibition is not yet known. Interestingly, quarfloxin and CX‐5461 are also two well‐known G4‐binding ligands that inhibit cancer cell growth through binding to G4‐structures and double‐strand break formation. Conversely, BMH‐21 binds to GC‐rich sequences and degrades the Pol I catalytic subunit RPA194 in cancer cells.^[36a]^ This observation supports our hypothesis that G4s might be also driving the inhibition of Pol I in *T. brucei*, where the binding of CX‐5461 to G4s would cause a block of the replication fork and induce DNA breaks (Figure 6). It is important to underline that no significant difference was observed between the *in vitro* anti‐trypanosomal activities of the three compounds, which suggests that Pol I inhibition is key for eliciting an antiparasitic effect, independently of G4‐stabilisation. Nevertheless, the greater selectivity and irreversible antiparasitic effect displayed by the CX‐5461 against *T. brucei* ^[36d]^ strongly suggests that G4‐ligands might be leveraged in the future for the treatment of neglected tropical diseases.

To assess how quarfloxin and CX‐5461 elicit their anti‐parasitic activity, G4 ChIP‐Seq and transcriptomic (RNA‐seq) analyses should be performed pre‐ and post‐treatment of *T. brucei* with the different drugs. This genomic investigation will generate a comprehensive map of G4s *in T. brucei*, while associating differentially expressed transcripts with G4‐formation. This will underpin potential transcriptional changes upon exposure to the G4‐ligands and unravel druggable pathways that can be further exploited for therapeutic development. Additional *in cellulo* validation may involve cellular imaging of pre‐ and post‐treatment samples, such as G4 immunofluorescence microscopy using G4‐selective antibodies (BG4).^[37]^ This would expand our understanding of (i) where G4s are located within the parasite's organelles, and (ii) determine what is the global effect of compound treatment on G4‐prevalence. For example, it is reasonable to expect changes in G4‐staining in the nucleoli of *T. brucei* upon treatment with quarfloxin and CX‐5461 if their mechanism of action is indeed mediated by ribosomal DNA binding. Conversely, no significant changes in G4‐staining should be observed with BMH‐21 given that its Pol I activity should not involve G4‐binding.

Given the importance of RNA Pol I in regulating the antigenic variation in trypanosomes through VSG expression, and that G‐rich telomeric sequences trigger the switching of the VSG genes,^[38]^ it is of fundamental importance to understand how G4s are involved in the activity of Pol I. As discussed in the context of the kinetoplast, we hypothesise that an analogous mechanism of action where G4s might be forming intra‐ and/or inter‐molecular structures to regulate the activity of Pol I is conceivable (Figure 6), especially considering that it has been demonstrated in human cells that multimolecular G4s, such as DNA:RNA hybrids, can interfere with the RNA polymerase complex and prevent the enzyme from progressing on to the template DNA strand by physically blocking the transcriptional process.^[39]^ Therefore, this G4‐mediated RNA Pol I inhibition mechanism can be leveraged as a useful chemical‐tool to explore the role of G4s in the activity of Pol I, and to drive the development of novel DNA‐targeted antiparasitic therapeutics.

## 5 Summary and Outlook

Computational analyses and *in vitro* biophysical assays have provided an overview of the prevalence, distribution, and stability of G4s, and they are useful for generating hypotheses on the role of these structures in regulating the transcription of genes involved in virulence pathways and host adaptation. However, the G4‐studies conducted so far in trypanosomatids are scattered in a way that they often include only a limited number of species or life‐cycle stages (*e. g*., insect form versus human bloodstream form) of the parasites. Therefore, to create a comprehensive reference G4‐map in trypanosomatid parasites, there is a need to employ genomic strategies in native chromatin across the different stages of the parasite's life cycles. For example, the previously mentioned G4 ChIP‐seq approach is pivotal to obtain a reference G4 map across multiple species and stages, and to potentially unravel the role of G4s as transcriptional modulators of key genes of potential therapeutic relevance, as discussed in this concept article. This technique utilises fixed and sonicated chromatin and, therefore, can provide a snapshot of parasitic G4s in cells. However, due to the chromatin being fixed at a specific stage of the parasite's life‐cycle, this method presents the limitation of yielding a static snapshot, where dynamics and real‐time interactions are missing. Thus, methods that utilize ligand‐mediated G4‐mapping in living cells would allow for highly dynamic single‐cell resolution. For example, G4‐specific fluorescent probes (*e. g*., silicon rhodamine‐labelled pyridostatin)^[40]^ would enable single‐molecule and real‐time detection of individual G4 structures in living cells.

Another caveat of the ChIP‐seq technique is the discrepancies in the number of identified G4s compared to the computational predictions (G4Hunter) and *in vitro* (G4‐seq) approaches. This has been observed in ChIP‐seq experiments in human cancer cells and it is attributed to the tertiary structure of chromatin being less accessible and, therefore, only a subset of the total G4s are revealed. Conversely, *in vitro* techniques, such as G4‐seq, are performed on linear and relaxed DNA strands, thus maximising the chances to detect any G4s that can be formed. This means that a higher number of G4s can potentially form within the parasite genome, but only a subset of them is biologically functional. Thus, identifying G4s that are detected in a chromatin context is essential to assess their potential biological relevance.

In this article, we have highlighted some recent work in the context of G4s in trypanosomatid parasites. The studies reported herein confirm that: (i) G4s are highly abundant in trypanosomatid parasites, with a distribution that is comparable to higher order level and evolutionary distant organisms, such as humans; (ii) G4s are not randomly located throughout the genome, but they localise at specific sites (*e. g*., gene promoters and transcriptional start sites), where (iii) they might regulate essential cellular processes that are key for parasite survival in the hosts, such as DNA replication and antigenic variation. Finally, we discussed an overview of chemical biology techniques that can be employed to unravel the fundamental role of these underexplored DNA structures and their impact on the genetic repertoire of parasites, thus presenting an exciting opportunity to advance research in neglected parasitic diseases with potential biological and therapeutic implications. Given the mounting evidence supporting the functional relevance of G4‐structures as epigenetic modulators in humans, and in light of the similar genomic distribution of these structures across the human and the *T. brucei* genomes, we anticipate that G4‐structures might represent a very promising avenue for therapeutic intervention against neglected tropical diseases.

## Conflict of interest

The authors declare no conflict of interest.

## Biographical Information

*Ludovica Monti is a Marie Skłodowska‐Curie Research Fellow at Imperial College. She obtained a PhD in Life Sciences (2018) from Sapienza University of Rome (Italy) and conducted part of her graduate studies in the USA. As a postdoctoral researcher and a member of the Center for Discovery and Innovation in Parasitic Diseases at the University of California San Diego (UCSD, 2018–2021), she worked on the discovery and development of new drugs for neglected parasitic diseases, namely schistosomiasis and human African trypanosomiasis. Her current research applies interdisciplinary sciences, including chemistry, biology, and genomics, to establish novel molecular approaches targeting the DNA of parasites*.

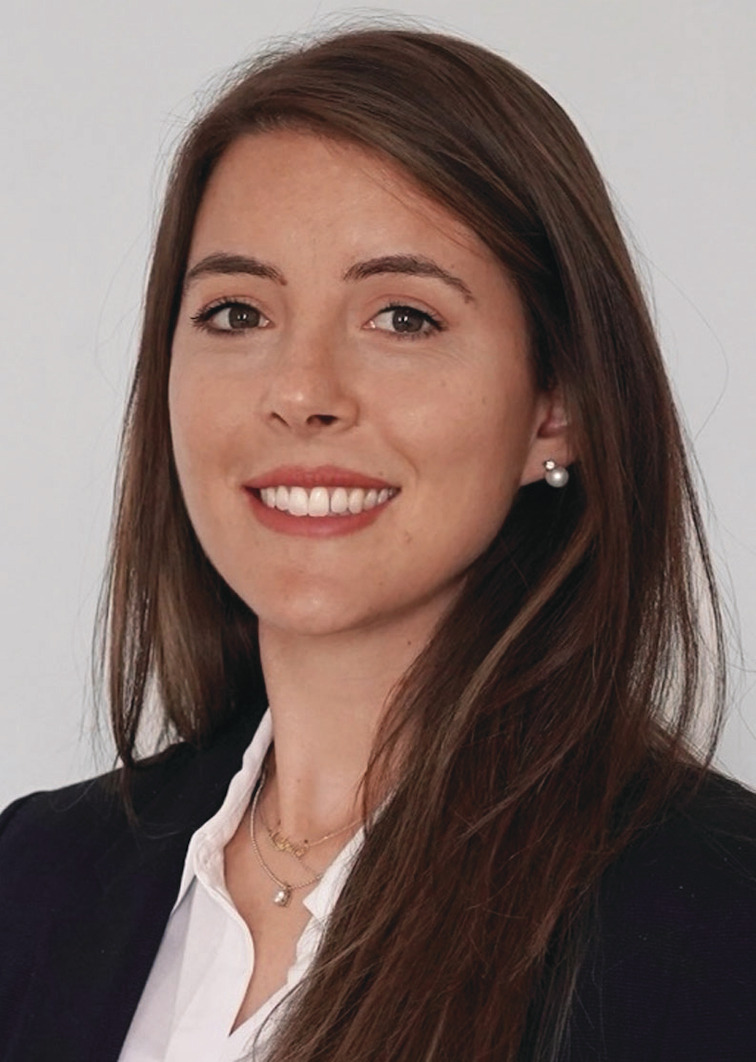

## Biographical Information

*Marco Di Antonio has a MSci in Chemistry from Pavia University (2007) and a Ph.D. in Molecular Sciences from Padua University (2011). He joined Cambridge University, as a Research associate in 2011, and continued as Senior Research Associate in 2015. He started his own group at Imperial College in 2018 as a BBSRC‐Fellow, before being promoted to Lecturer in 2022. He holds a satellite group at the Crick Institute and a Lister Research Prize (2022). His current research focuses on combining chemical biology tools with genomics to investigate the role of nucleic acid structures and modifications in aging and cancer biology*.